# 533 Control Acute Klebsiella Pneumoniae Infection in Burn Wounds with Acidic Cream

**DOI:** 10.1093/jbcr/iraf019.162

**Published:** 2025-04-01

**Authors:** Ping Chen, Andrea Fourcaudot, Eliza Sebastian, David Silliman, S L Rajasekhar Karna, Johnathan Abercrombie, Kai Leung

**Affiliations:** US Army Institute of Surgical Research; US Army Institute of Surgical Research; US Army Institute of Surgical Research; US Army Institute of Surgical Research; US Army Institute of Surgical Research; US Army Institute of Surgical Research; US Army Institute of Surgical Research

## Abstract

**Introduction:**

Burns compromise the skin’s natural barrier function, rendering burn wounds susceptible to microbial colonization and infection. Wound infection is the most common complication of burns, highly correlated to the mortality and morbidity, and is the major cause of death in burn victims. Klebsiella pneumoniae, a gram-negative bacterium, is one of the most predominant wound pathogens in burn infections. K. pneumoniae colonizes burn wounds and disseminate systemically, leading to invasive infections and septic complications. In addition, K. pneumoniae is well-known for its ability to acquire antimicrobial resistance through various mechanisms. This ability reduces the effectiveness of commonly used antibiotics in treating K. pneumoniae infections. Therefore, there is a compelling need for alternative antimicrobial agents or combination therapies for treating Klebsiella infections. We report the use of an acidic cream to control acute K. pneumoniae infection in burn wounds.

**Methods:**

Acidic cream was prepared using the active ingredient sodium diacetate. An ex-vivo-burned porcine skin wound model was used to determine the effectiveness of the acidic cream against K. pneumoniae. In vivo activity was evaluated using a modified Walker Mason rat model of deep partial-thickness dorsal scald burn. All rats were anesthetized with isoflurane and analgesia with BuprenorphineSR was provided prior to and post all procedures. The treatment effect was assessed by comparing the viable counts of K. pneumoniae recovered from treated and untreated (base cream) infected burn wounds. Additionally, tissue cytokine levels, tissue myeloperoxidase activity and the pH of the burned skins were determined.

**Results:**

In the ex-vivo-burned porcine skin wound model, the acidic cream significantly reduced (approximately 9 log) K. pneumoniae from the infected burn tissue 24 hours post-treatment. In the rat model, after two daily treatments, the acidic cream treatment reduced the Klebsiella viable counts by approximately 4.2 log. The pH of treated wounds decreased to approximately 6.1 as compared to 7.1 of the untreated wounds. Compared to the base cream treated, cytokines/chemokines of acidic cream-treated tissue which included MIP-1α, Gro/KC and IL-6 levels were significantly elevated. The myeloperoxidase activity in the treated burn tissue were also higher.

**Conclusions:**

Sodium diacetate-based acidic cream could effectively control acute K. pneumoniae burn wound infections.

**Applicability of Research to Practice:**

To provide an alternative to the traditional antibiotic treatment of acute K. pneumoniae burn wound infections thus minimizing emergence of antibiotic resistance.

**Funding for the Study:**

Studies are supported by the Congressional Military Dental Research Program administered by the Naval Medical Research Command Naval Advanced Medical Development

